# Multilevel determinants of paternal and child physical activity: qualitative research using dyadic interviews among Mexican heritage fathers living near the Texas-Mexico border

**DOI:** 10.1186/s12889-025-23956-x

**Published:** 2025-08-16

**Authors:** Marilyn E. Wende, M. Renée Umstattd Meyer, Serena Enriquez, Christina N. Bridges Hamilton, Tyler Prochnow, Joseph R. Sharkey

**Affiliations:** 1 Department of Health Education & Behavior, College of Health and Human Performance, University of Florida, FLG 73, Gainesville, FL 32611 − 8210 USA; 2 Department of Public Health, Robbins College of Health and Human Sciences, Baylor University, Waco, TX USA; 3 Department of Kinesiology, College of Health and Human Sciences, Kansas State University, Manhattan, KS USA; 4 Department of Health Behavior, School of Public Health, Texas A&M University, College Station, TX USA

**Keywords:** Father, perceptions, Qualitative, Physical activity, Environment, Dyadic

## Abstract

**Purpose:**

Families living near the Texas-Mexico border face disproportionate barriers to physical activity (PA), yet little research has explored how Mexican-heritage fathers perceive and overcome barriers to child PA. The purpose of this study was to examine and describe fathers’ perceptions of strategies to improve child PA through a social-ecological lens.

**Methods:**

Fathers (*n* = 30) living near the Texas-Mexico border *colonias* completed Spanish-language, father-father dyadic interviews (*n* = 15) conducted by trained facilitators. Spanish-language audio recordings were transcribed verbatim and translated into English. A coding framework was created based on the social-ecological model. Inductive and deductive approaches directed thematic analysis. Coding consisted of two researchers who coded one interview for reliability purposes with intercoder agreement set at 80% agreement on 95% of codes.

**Results:**

Fathers mentioned multilevel barriers to PA, and outlined strategies that demonstrated their resilience in promoting PA among their children. Participants discussed intrapersonal factors (e.g., physical health, weight status) as barriers to their child’s PA, but their experiences with their own health issues motivated fathers to promote healthy behaviors in their children. Interpersonal factors (e.g., parental duty, social support, culture) facilitated PA, and were a major way fathers overcame unsafe outdoor environments to facilitate PA. Community and family supportiveness and closeness were cultural norms that were crucial for facilitating outdoor PA. Many fathers reported working long hours and having low access to well-paying jobs as a major barrier to PA. Given that fathers often came home from work late, participants developed creative solutions to facilitate outdoor PA, like installing lights to play outside at night. Finally, participants reported finding meaning in PA for themselves and their children, including connection with family and friends, culturally relevant activities, personal identity, and health maintenance.

**Conclusions:**

The results of this study suggest that the meaning participants attribute to being physically active, especially on the interpersonal level, contributes to reinforcing PA and promoting resilience for themselves and their children. These findings can inform the design of culturally grounded interventions that leverage fathers’ interpersonal strategies and resilience (e.g., family-centered activities, addressing neighborhood safety) to support PA among children in low-resource, Mexican-heritage communities.

**Supplementary Information:**

The online version contains supplementary material available at 10.1186/s12889-025-23956-x.

## Introduction

Less than half of United States (U.S.) children and adolescents meet physical activity (PA) guidelines [1], and inadequate PA is linked to an increased risk of adverse physical health outcomes [2, 3]. Decreasing rates of PA and increasing rates of sedentary behavior among children and adolescents indicate that more directed strategies are needed to promote PA [4–9].

Previous research has shown that parents are instrumental for child PA through logistic support [10], modeling [11], social support [12, 13], and co-participation [14, 15]. Research shows that fathers’ perceptions of PA opportunities and barriers greatly influence their children’s PA outcomes [11, 15–21]. Fathers hold a distinct role in PA promotion, by modeling PA behavior, instilling cultural expectations, and upholding parental roles to facilitate PA [11, 15, 22]. Research is also needed focusing on Mexican-heritage fathers who are at higher risk for physical inactivity, may face unique barriers to promoting PA among their children, and have a unique role in PA promotion [23, 24]. While an increasing number of physical activity promotion efforts are family-focused, there has been inadequate involvement of fathers [25, 26]. In addition, little is known about paternal perceptions of more social determinants (e.g., policies, environments) of PA and successful strategies for overcoming barriers to PA [11]. Understanding fathers’ perceptions of multilevel PA influences is critical for developing culturally relevant, family-centered interventions that equitably engage Mexican-heritage families in physical activity [27].

A specific subpopulation that faces marked place-based barriers to PA participation and chronic disease prevention resides in marginalized and often unregulated neighborhoods on the border of Texas and Mexico– referred to as “*colonias”* [28–30]. People living in *colonias* are classified as systematically underserved due to their lack of access to basic infrastructure (e.g., drinking water, paved roads), high levels of persistent poverty, and disproportionate rates of chronic conditions [29, 31–33]. *Colonias* have few PA resources, such as parks [34, 35], and face barriers to active transportation (e.g., limited sidewalks, few safety features) [36]. High temperatures in southern Texas Create additional barriers to PA, and parents often have to work long hours [37]. Adding to this, insecure immigration status shows a negative influence on PA and other health behaviors [38]. As a result, those living in *colonias* show low adherence to PA guidelines: 67.6% of respondents did not meet PA recommendations [39]. Despite these challenges, *colonias* show many assets. *Colonias* residents demonstrate high levels of mutual, social support between family and community members, which is instrumental for overcoming the negative consequences of poverty [40]. Residents view their colonia as a stable, long-term community where multigenerational Mexican-heritage families build lives, invest in one another, and envision a future where they can raise children, thrive, and age with dignity [40].

To collect information on paternal influences on and perceptions of child PA in Mexican heritage families, dyadic interviewing is an underused strategy. Dyadic interviews are a qualitative research method where two participants participate in dialogue in response to open-ended research questions [41]. Dyadic interviews facilitate interaction and comparison between participants similar to focus groups, but involve only two participants while focus groups involve groups (i.e., 6–8 people recommended) [41, 42]. Dyadic interviews have been identified as a strategy for qualitative research with families since they offer insight into how family members might respond or react to each other in less formal interview settings [43]. Although some PA research has used dyadic interviews to study father-mother or father-child pairs [44–48], dyadic interviews could also be used to uncover the common experiences and reciprocal dynamics between father-father pairs. Father-father pair dyadic interviews may offer an opportunity to share cultural experiences between male peers, and facilitate emotional expression that is less prevalent among male participants in qualitative interviews [49, 50].

In summary, there is limited understanding of paternal perceptions of barriers to family PA, particularly how fathers interpret and navigate multilevel influences—including individual, social, environmental, and policy-level factors. Adding to this, research is needed to examine the barriers and facilitators to PA in *colonias* and similar under-resourced settings, with particular attention to the role of social support and how it interacts with broader structural and environmental influences. Therefore, the purpose of this study was to examine and describe Mexican-heritage fathers’ perceptions of strategies to improve their children’s PA by applying the social-ecological model. Results from this qualitative study using dyadic interviews can inform future initiatives in similar communities by identifying contextual barriers and potential ways to improve access to and utilization of PA opportunities near the Texas-Mexico border.

## Methods

### Sample

*Promotoras de salud* (trained female community health workers) [51] recruited Mexican-heritage fathers (*n* = 30) residing in geographic clusters located near unincorporated San Carlos, Texas (along the US-Mexico border in Hidalgo County, Texas) to complete dyadic interviews (*n* = 15 dyads) to inform a culturally tailored nutrition and physical activity pilot program [23, 52–56]. A total of 15 dyads (30 participants) were included due to timing. To select these geographic clusters, *promotora* researchers led formative work within 18 total geographic clusters in Hidalgo County, 12 of which were in the San Carlos area [23, 52–56]. Inclusion criteria required fathers to be of Mexican-heritage, live in Hidalgo County for at least one year, have at least one child between 8 and 10 years old, prefer written and oral communication in Spanish, and reside in the same household with their partner and at least one child 8–10 years of age. Fathers were not excluded if they had any PA restrictions, and we did not limit the involvement of fathers with disability in this study. Fathers were paired based on availability, and were unknown to their dyadic pair.

### Study setting

Hidalgo County, Texas has over 860,000 residents of which 91.8% identify as Hispanic [57]. Median household income in Hidalgo County was lower than state levels ($37,106 versus $59,206), and use of the Supplemental Nutrition Assistance Program is more than double statewide levels (30.5% versus 12.0%) [57]. The U.S. Department of Health and Human Services has identified Hidalgo County as medically underserved and under-resourced due to its increased social and health barriers, high poverty rates, and disproportionate rates of disease [31, 58].

### Data collection

Dyadic interviews were conducted at a local church and community center to ensure privacy and increase fathers’ comfort levels [41]. After pilot testing the procedures [52], two trained facilitators (i.e., research staff and/or a *promotora de salud* trained on approaches to engage Mexican-heritage fathers in discussion) sat at a table with two fathers in chairs positioned across from each other at the table. Fathers were seated across from one another to increase interaction and the flow of discussion in a way that resembled conversation. The facilitator was provided with a Spanish language script including instructions that they would only speak to introduce the next topic or respond to questions presented by the fathers. The facilitator also asked fathers to introduce themselves, and discuss family, hobbies, and employment. This was followed by open-ended discussion-based prompts. All materials and protocols were approved by the referent institutions’ Institutional Review Boards. All participants provided written informed consent prior to enrollment in the study.

Paternal dyadic interviews consisted of open-ended prompts developed with iterative feedback from *promotoras de salud* or research staff (see Additional Files 1 and 2). Follow up prompts were created to augment discussion of perceived influence and responsibilities for PA, nutrition, technology use, and general activities in which the fathers regularly participated. Examples of follow up prompts included: “Tell me about your role as a father”; with prompts including: “what do you do as a father?”, “what does it mean to be a father?”, “what are your responsibilities at home?”, and “what is most important about being a father?”

Reported child PA and paternal role in child PA were prompted with two questions: (1) “What do you think about your child’s PA” and (2) “When you think about how your child plays and is physically active, how much of what they play or do is influenced by you?”. Question 1 follow up prompts included: “Why do you agree or disagree with their physical activities?”, “Regarding PA, what would you prefer for them to do?”, “How is it different or similar for your other children?”, and “Is it similar or different to what you have experienced?” Question 2 follow up prompts included: “Others in your family?”, “Do you have rules?”, “Are there any physical activities or active play that you do with them?”, “Do you do different physical activities during the week versus the weekend?”, “What are some of the difficulties of being physically active with them?”, “Are there any house responsibilities they help you with?”, “How is it different or similar to the way you influence your other children?”, and “Is it similar or different to what you have experienced?”

### Researcher positionality

This study was conducted by a diverse team of researchers and students, several of whom had spent substantial time in colonias and developed close working relationships with participating families. Promotoras de salud—trusted health promotion specialists living in colonias—led all interviews and played a central role in shaping the research process. The coding process was led by a White woman researcher with expertise in social and environmental determinants of health. Although not a resident of colonias, the analysis was guided by a commitment to centering community perspectives and engaging in reflexive, team-based interpretation. The coding team included a woman who resides in a colonia and whose family members also live there, providing valuable insider insight during analysis.

### Analysis

Descriptive statistics were used to provide demographic information of participating fathers including mean age, paternal education (less than high school, high school diploma), country of birth (United States, Mexico), and employment status (unemployment, part time, full time, or both part and full time).

Spanish-language audio recordings were transcribed verbatim and translated into English using a four-step transcription and translation process to ensure completeness and accuracy of transcription and translation. Transcripts were coded using NVivo software. A coding framework and operationalization table were collaboratively created (MEW, MRUM, CNBH, TP, SE) based on the social ecological model [27, 59].

The coding process was collaborative, iterative, and included peer review for maximum quality. A combination of inductive and deductive approaches were used for the thematic analysis of codes [60]. To address potential research bias, coding consisted of two researchers (MEW, SE) who coded one interview for reliability purposes with intercoder agreement set at 80% agreement on 95% of codes. Researchers independently coded the remaining participants and then combined codes into common sentiments in an initial hierarchy. Finally, a conceptual framework was created based on the social-ecological model to reflect and capture the ways in which multilevel themes were overlapping. The conceptual framework was created to reflect the coding framework as it was developed and was refined throughout the coding process. To ensure data quality, coding included utilization of the framework coupled with memos (researcher-developed insights), diagramming (quote selection for father-to-father comparison), categorization, and topic monitoring (parallels between fathers) to complete paternal profiles [61, 62]. Table 1 provides a full list of codes and related descriptions.

**Table 1:** Coding framework

**Ecological Model for Active Living–** ***Policy Environment***	
Public infrastructure investments	Discussion about layout/infrastructure of *Colonias* or Hidalgo County, discussion of investment in community resources, such as parks, recreational facilities, walkable spaces, or “free fun”.
Zoning & developmental regulation	Discussion of gentrification, development of certain areas (i.e., new suburban or commercial areas being built acting as barriers or facilitators to PA), suburban sprawl characteristics, ability/inability to create homestead due to zoning policies, or barriers/facilitators to living in a more desirable/safer neighborhood.
Law enforcement policies and funding	Discussion of reasons or policies behind police surveillance, law enforcement funding, or existing law enforcement initiatives (e.g., war on drugs, reduce property crime, increase neighborhood safety, deportation efforts). Discussion of barriers/facilitators doing certain activities due to immigrant status and related laws.
School or education policies	Discussion about access to quality schooling, good/bad influence of certain physical education policies, or school policies that act as constraints or facilitators to physical activity.
Parking and traffic investment/planning	Discussion about road types (e.g., highways, fast roads) close to home environment or access to parking near physical activity resources (e.g., parks).
Access to jobs	Discussion of access to quality jobs, notes about only job opportunities within *colonias* entailing long and laborious commitments, or talking about needing to travel to other places for work that matches skill set.
**Ecological Model for Active Living–** ***Physical Environment***	
Neighborhood physical activity opportunities	Parks, facilities, aesthetics, places to fish, soccer fields, free recreational classes/centers, after school-programs for children, etc. Neighborhood is defined as environments outside the home/yard, that are considered nearby or convenient.
Play and activity opportunities in the home	Ability to do physical activity in indoor spaces, physical activity using recordings on TV or stereo, breakable material in the home, need for quiet in the home, etc.
Play and activity opportunities around the home	Yard space, play equipment in the yard, features in the yard that require active labor (e.g., farming, chickens), etc.
Climate	Weather events, rain, heat, cold, ice, etc. that serve as barriers or facilitators to physical activity.
Safety	Discussion of traffic safety or crime safety in environments with which participants interact. Discussion of places or spaces deemed “safe” for PA, such as stating that they only feel safe being active at home.
Surveillance	Discussion of police surveillance, surveillance by other residents when using community resources, cameras in community spaces, etc.
Relative deprivation	Discussion of high poverty levels in community, access to free activities or certain outlets nearby, maintenance of community resources, etc. in comparison to other communities.
Sedentary opportunities	Discussion of access to video games, television, cell phones, iPad, etc. or places to go that entail sedentary behavior (e.g., movie theatre).
Access to transportation	Discussion of access to public transport, access to vehicle, ride sharing, transportation as a barrier/facilitator.
**Ecological Model for Active Living–** ***Interpersonal***	
Community	Community defined as “a group of people living in the same place or having a particular characteristic in common”. Nearby friends or acquaintances, either through school, work, neighborhood, or other setting. People who provide emotional or instrumental support to fathers or their families. NOT including family members (community = people outside family).
Family	Characteristics of participant’s family noted, such as family structure & size, extended family interactions/dynamics, importance of family.
Culture/norms	Mention of holidays, church, soccer, fishing, cultural resistance, traditions, etc. that relate to cultural background. Norms in the United States, norms in Mexico, gender norms, adherence to norms in general and/or around physical activity (e.g., gender norms for fathers and children for what PA looks like).
Parental and caregiving roles	Participant’s perceptions of their parental and caregiving duties and roles in the parenting/caregiving process, including caretaking, modelling, quality time, teaching, discipline, etc.
Occupational roles	Participant perceptions of their role as a laborer: physical labor at work, long hours, long way to travel for work, relationship with employer.
Household roles	Participant’s perceptions of duties to complete chores/cleaning, take care of yard/homestead/animals, decorate, make food, etc.
Social support	Mention of general social support, familial social support, friend social support, social support for PA, etc. Could relate to support given by the participant to others or given by others to participant (e.g., social support received by or provided to children, partner, family members, friends).
**Ecological Model for Active Living–** ***Intrapersonal (in Nvivo as “Participant”)***	
Recall	Feelings/emotions/impressions around childhood/past experiences that still impact life (e.g., trauma, nostalgia) or past experiences with PA.
Obligation	Feelings/emotions/impressions around expectations of them (e.g., guilt, confidence). Feelings that they must put personal needs/interests aside to meet expectations. Could present itself as a positive emotion, as well, if participants rise to opportunity (e.g., use need to do chores as opportunity to engage kids in physical activity).
Physical health	Feelings/emotions/impression on relationship between physical activity and physical functioning (bi-directional).
Mental health	Feelings/emotions/impression on relationship between physical activity and mental functioning (bi-directional).
Beliefs	Discussion of spiritual beliefs, guiding tenants, values, ideas about or importance of PA, etc.
**Additional themes**	
**Resilience**	[Major theme to identify] Relate to strategies for overcoming/coping with barriers or taking advantage of opportunities for physical activity. May provide a connection between more distal influences and individual meaning/experience of physical activity. *How do participants conceptualize their way of adapting to circumstances to maintain physical activity?*
**Meaning/Experience of physical activity**	**Outcome**
Intrapersonal/participant	Physical activity as beneficial/detrimental to physical & mental health, physical activity contributing to sense of self, physical activity as a way of dealing with past experiences (e.g., trauma), physical activity as a way to deal with feelings of obligation (as parent, in household, etc.).
Interpersonal	Physical activity as a way to model/teach good behavior to children, bond with family or community, participate in cultural resistance, connect with others, fulfill job tasks, etc.
Environmental	Physical activity as a way to connect with nature, take advantage of neighborhood resources (e.g., free fun), get from place to place or travel, deal with climate conditions, etc.
**Negative**	**Negative code will be used to code anything within the other codes that is negative.**
**Positive**	**Positive code will be used to double code anything that is positive within the other codes.**

## Results

Fathers within this study had a mean age of 41.2 years (SD = 9.6); most were born in Mexico (*n* = 24, 80.0%), employed full-time (*n* = 17, 56.7%), and did not complete high school (*n* = 21, 70.0%). Fathers mentioned policy, environment, interpersonal, and intrapersonal level factors influencing their own and their children’s PA.

### Conceptual framework

**Fig. 1:**
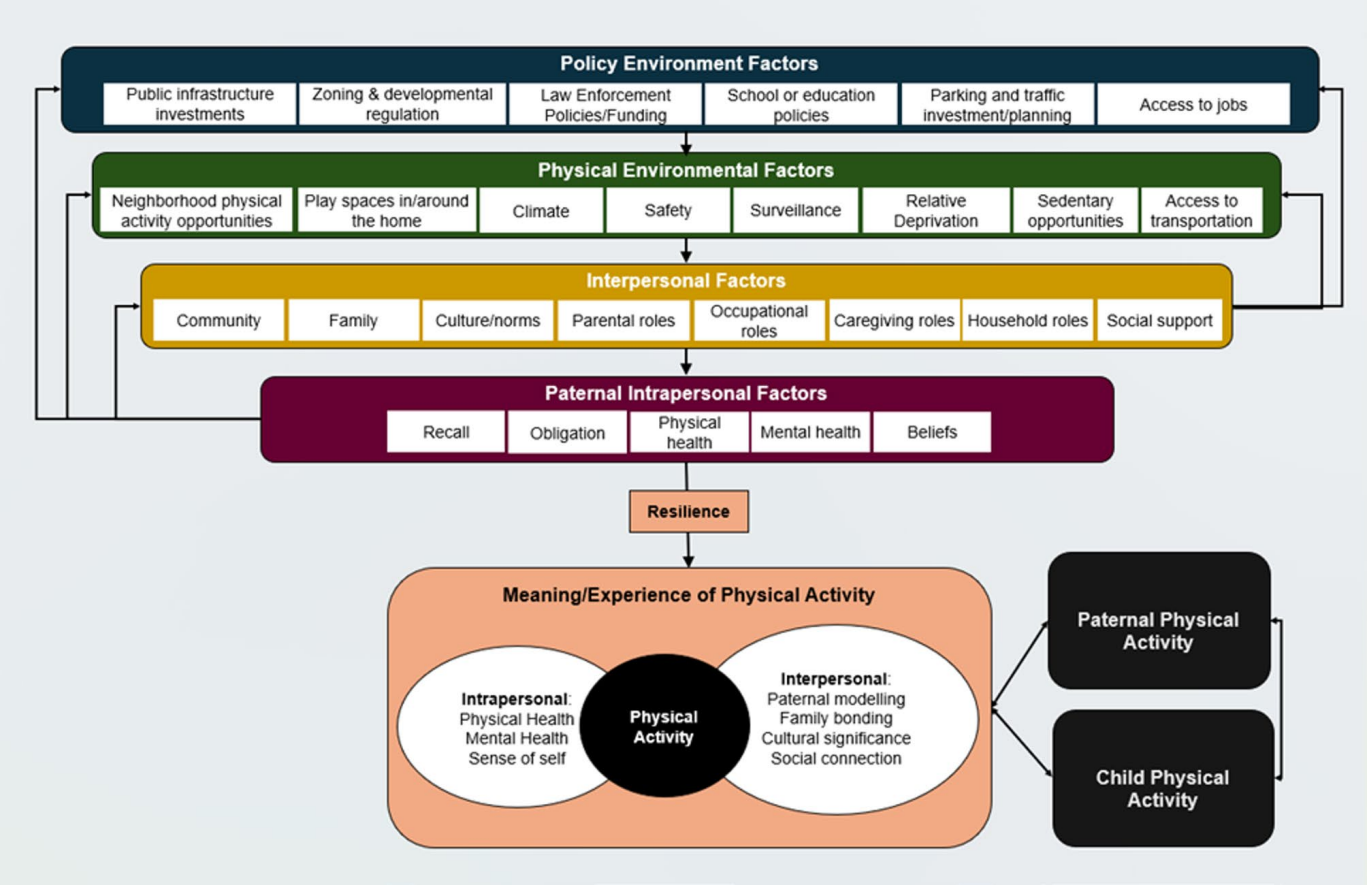
Conceptual framework

Figure 1 reflects the interactions between policy environment, physical environment, interpersonal, or intrapersonal level themes expressed by fathers in this study. Environmental and policy-level barriers were often addressed using interpersonal support systems. Intrapersonal-level paternal beliefs, recall, and physical and mental health were also strong motivators for fathers to engage in PA with their children (Fig. 1). Resilience, defined as positive adaptation despite adversity, was demonstrated by fathers to overcome the myriad policy environment, physical environment, interpersonal, and paternal intrapersonal level barriers to PA they faced [63]. Fathers expressed that PA held great meaning in their lives in its ability to promote health, develop their sense of self, fulfill their role as fathers, create opportunities for culturally significant activities, and connect them with their local community and nature.

### Policy influences on PA

Although policies were not frequently discussed in relation to PA, participants did outline how some distal factors influenced their own and their child’s PA. Policies are less changeable or under the control of the fathers in this study, but had a profound impact on PA opportunities and resources.

Parking, traffic investment, and planning policies were important for child PA, given that traffic was a major safety concern related to playing outdoors and many fathers reported that they lived near busy roads:

I don’t like to leave them outside because, since it’s not fenced in or anything… you just had a distracted [moment] and the kid is going to the road and well, there they do drive fast. And that’s why just while I’m outside, then when I go inside, I bring them in with me. But I don’t like them, them to be there alone there.

Fathers discussed providing supervision, enforcing strict safety rules, providing transportation to safer PA opportunities, or redirecting children to play indoors as adaptive strategies to overcome traffic and planning barriers to PA (Table 2).

**Table 2:** Examples of coded themes from dyadic interviews among Mexican heritage fathers in Hidalgo county, Texas (*n*=30)

Theme	Example
**Ecological Model for Active Living–** ***Policy Environment***	
Public infrastructure investments	**[ID #22]**“That, what’s more, even in Mexico that is a really very poor place, you still see {soccer} fields. Places where you can get distracted. You see a lot, a lot of activity from the kids… compared to what we see here, uh, it’s nothing not even close… Whether it’s the, the park, or if it’s in the {track} of the school but we are saying, only in that it will take fifteen minutes to [get] there, fifteen minutes to [come] back… In these places of this, this area, here in the north because of the amount of population there is, there’s nowhere to take the kids that you say, “You know what? It’s nearby.””
	**[ID #22]**“When we were little we didn’t have a park like that but they were climbing up and down from trees, uh, on the fence, above the house. Uh, doing things like that and, and, and it’s something that all of them love, that as they’re growing up and they’re older, you’ll see them where, you know what? They want to play a sport, they want to play {soccer}, they want to play {basketball}, that park is even more useful for them, but there aren’t enough for everyone. It’s like they say, they either do [things] in this park in this way or they do [them] in another park and to go to one of those that have space you go to, to [nearby location] or you go to, to the ones in [different nearby location] that actually have more space. And you get there too and what’s interesting is that you get to those parks, there’s no one. There’s no one… And you see them in smaller places or in towns or, or where it’s more urban here that, the park is not that big or that and it’s full because the people that, that’s trying to go to those parks where they are, they’re not in the parks.”
Law enforcement policies and funding	**[ID #02]**“They’re scared. I, since I already go out with them, I go, I leave, I go. Not long ago I took just the oldest ones on vacation to {Georgia}, my wife can’t leave because she is working on her papers. She asks permission to be here but not to leave the, that is, from the garita. {So} then I, her, her children, don’t have papers, her two, the older ones. I worked it out, we all went to Juarez, no ~ a year ago. That was a year ago, I sorted out their papers. They’ve had their papers worked out for a year. The [male] sixteen-year-old and the [female] that ~ that is going to turn, uh, fifteen. I went over to Juarez with them, just me and those two, I went over. After fifteen days we came back. There’s not much, I took my oldest son, the sixteen-year old, the fifteen-year-old girl, and my son who’s five. We went for a week and a half. I spent [it] with relatives in {Georgia}… I couldn’t take my nephew with me of, he doesn’t have papers. He’s here right now, well he’s just in school. But my wife wants to get him a permiso but there wasn’t, wasn’t time and I just went like that. But I did, did, did take him, {I mean}, we do, do go out and around, and when there’s a chance we go anywhere [around] here.”
	**[ID #16]**“I always have those rules like, “Go and play, go and play outside where I see you. And outside, ~ not in the street.”… Because I’ve already had problems because of the same thing there where the police pass by a lot and one time they passed and saw a ton of kids and I was the one who took her because I already had a problem before. Where I used to live and they even came down on me, it all came down on me. They even wanted to take my children away because they were out playing on the side like that. ~ well it’s that the yard is like that, I said, just that, well, it was bad timing how they passed and looked there, well, they said that they didn’t see anyone there, well we were like that sitting very close to the door, and you couldn’t even see. And that’s why since then I have only, a lot of rules with them in the street.”
School or education policies	**[ID #09]**“I remember that when they had the {open gym}, they would open the school gm as well and, I didn’t, in reality I don’t know why they don’t do it anymore, because it’s a public school. They should do it by law, but they don’t, they don’t do it. And I remember that before, well my dad wasn’t really ~ of that, but I remember that when I was ~ well, they would open the gym and they would open that one and I would take advantage and go.”
	**[ID #11]**“Sometimes when, at first I would let him play a lot, imagine, and suddenly he, I calmed him down. Then I didn’t let him play any activity, nothing, not even at school. Because, supposedly they had diagnosed him that he had, what’s it called? Asthma, and they even had him with that, I told him, “Man, he there’s nothing wrong with him, there’s nothing wrong with him.” And he said, “No, yeah because…” I took him to the doctor once, I took him when his throat hurt him, and then they diagnosed him with that, they said, they gave me a paper for him to take to school, so that he wouldn’t have any activity, for him to just be sitting down. No, well, after that no, I said, “Man, there’s nothing wrong with him,” and one time I said, “Let him try it.”
Parking and traffic investment/ planning	**[ID #25]**“Yeah the other neighborhood yeah, no, we don’t let her go because, but also because she goes further away ~ back. So I say, ‘No, don’t be going so far because that is dangerous, dangerous crossing the street and all that.’”
	**[ID #20]**“Uh, where he can’t play outside, well, it’s in the, like I said, there’s an entrance, keep in mind, it’s just an entrance, a lot of cars are coming in the, he knows that there in the street, he cannot play where the street begins. Uh, inside yes, but he cannot play there in the street. For the same reason, right, that sometimes a few [cars] drive-in fast, uhm, they go after the ball and they’re not paying attention, they’re going to get in the way and there goes an accident. But he knows that he can play wherever he wants there inside, but not outside in the street.”
	**[ID #10]**“When they see that I’m already outside, well, they want to go outside. I don’t like to leave them outside because, since it’s not fenced in or anything, it’s all open, so that it doesn’t happen that he, you just had a distracted [moment] and the kid is going to the road and well, there they do drive fast. And, uhm, that’s why just while I’m outside, then when I go inside, I bring them in with me. But I don’t like them, them to be there alone there.”
Access to jobs	**[ID #13]**“Uh, cutting asparagus and putting it in little boxes and…it works well for us. Like, you can make some good money, sometimes it’s a thousand per week. But, it’s only six weeks, the four weeks it does, does go well for you. The other two it’s already, about to end. But, well, yeah, you do what, pretty much here you make in a whole year [is] what you make in six months there in Michigan. {So}, that’s why we go there.”
	**[ID #01]**“No, well, that is the responsibility, like he says, of each one, of the head of the house, you know what I mean? I worked years ago, I worked when I was in the {Border Patrol}. The ~ was 24 h that I had to work and sometimes I didn’t get [home]. And sometimes I got used to not even see my wife, or the kids because, well, there wasn’t time, they went to school. But at the same time you lose that affection with the kids, you lose that opportunity to get them, to take them to play or to eat together and all that. Many opportunities are lost.”
	**[ID #08]**“We left, we left up north the first year that I put [papers for] my wife and my son in order, because it’s been five, six years since I set them in order. And I took them both up north. We all left and I say, “Come mijito, so that you see how money is earned.” “So that you learn how, what I suffer.” Not what, because it’s been a long time since I’ve had [my papers] in order. I sa-, “Just so that you see how I suffer to earn money.” No, well, you have it there, he doesn’t want to work anymore, he only worked one year. Because he says that the work is really hard and I told him, “And you imagine, I would come here and you’re like, ‘Send me this, send me that.’ And you didn’t even know how I earned it. Now you do know how I earn it, right, because your back hurts, everything hurts.” I told him, “That’s so that you see.” “There it is,” I told him, and everything that pe-, demanded there, now, not anymore.
**Ecological Model for Active Living–** ***Physical Environment***	
Neighborhood physical activity opportunities	**[ID #03]**“I think that she kind of feels like she isn’t, like exercise is not for her… but she likes running, she likes to go out to play. When we used to live in the apartments, there’s a lot of, like you say, there’s a lot of kids that go and play and, and many of those kids are the kind that, that the moms or dads don’t, don’t take care of them. So since it is an apartment and it’s enclosed, well, the whole afternoon since they get out of school, they are up and down and it gets dark and those kids are still outside.”
	**[ID #05]**“Yes, because it makes them really, really happy that you’re with them. Because they trust you. They trust you. With them I, until now, I get along with them very well, with the one in the middle, the oldest, and the little girl. With everyone. The oldest, he gets along right away. “Let’s go play.” “Fine.” And the, the middle [female] child plays fútbol a lot. We go to the park ~ we play fútbol. But yeah, they trust you, as, as a friend, she feels like you’re ~ playing with her they tell you everything.”
	**[ID #07]**“No, well we go to the park when they, well sometimes we go once a week. It depends on how they’re doing when they get out of school, because sometimes they get home tired, whatever and, or sometimes they tell me, “Daddy, let’s go to the park.” “Right, well let’s go for a while because it’s late,” but, when we can, when we have enough time, “Alright, let’s go to the park.” Sometimes I take them to the park for two hours. And then, well they play what they want.”
	**[ID #08]**“But it’s what she does, sports more, she likes soccer. Ah, and riding her bicycle, around, around there, in the colonia. Since there are no cars there, there isn’t anything, there they can as-, she takes out the bicycle and goes around with everyone there. Everyone has their little bike there and it’s all that my daughter does is all, but not, just more, more drawing. It’s her sport. Well, it’s also a sport for her because she likes it a lot.”
Play and activity opportunities in the home	**[ID #16]**“Well, my daughter… she likes is dancing. She likes Zumba. Her mom has Zumba videos and she likes it. She [has], I bought her a little speaker for the music and she puts it on {Bluetooth} and then she puts on just music for, to dance and she says she’s going around dancing Zumba there with the girls, her [female] friends from the… And that is what she likes most because, like playing something else, no. Just dancing. Dancing and playing, well, like children’s games chasing each other around like that.”
	**[ID #09]**“Because when my wife is there alone, well she says, “Man, to have them there outside.” “Some take off that way to the street and others the other way and I can’t watch them anymore.” And that’s why no, she doesn’t take them out, but there inside the house keep in mind that they have a track. From some rooms to another and then back.”
	**[ID #08]**“But, it’s what she helps me with, but the little girl, the two year old baby grabs the broom and the dustpan and she goes and sweeps the whole mess that I make. The little girl, I tell her, “What your mom didn’t do,” I told her, “who is all grown up.” She is two years old, my daughter, she already can [use] the broom and dustpan. And she goes and helps me pick up all the tra- [trash], because we’re throwing down a lot of trash.”
Play and activity opportunities around the home	**[ID #05]**“Yes, we get [home] later at night, because, the night falls. But when I am with what I do, and there are times when we are, there outside of the house, like the ~ gentleman says, that we pick up everything that’s on the floor, the trash, everything, we clean the yard, clean the house. So we are, ~ all of us cleaning there. And they helped inside. I have three girls. ~ two older ones and a little one. The oldest help mom inside and the boy here outside with me, the other one. He’s thirteen already, {so} he’s picking up [stuff], he mows the lawn, cleaning. And he helps when I do jobs there at home, mechanic [work].”
	**[ID #08]**“She has her trampoline outside there, she also goes out to jump there a lot. Him and the, her and the girl. And she really likes for me to play with her. With both of them there, they want me to tickle them and tickle them, and they never fill up, never.”
	**[ID #09]**“I try to, uh, when I get out early, I try to, it’s that, I just bought a plot of land in, in the property where we are now further back and sometimes in the evening I arrive and I go back and I’m cleaning, because it’s forested, and they love to be back there. What I did, uh, I bought them a little golf course1 so that they start to play back there. I said, “Well now that, that…we have to give them time,” right? But also, it worries me that, at the same time to play with them and, and having to do what… They see it as a game, but, well, in reality I am cleaning and at the same time…they tell me, “Look, dad, the ball and that…!” No, ~ I, uh, also when I get home early it’s for that. I try to, sometimes not to, I’m showering at ten, eleven at…because I get there and they’re waiting that, they want to go ou-, out, to the bikes to…and I there I go out because, well, because we can’t (Laughs) leave them alone there. Man, they pass by very (Laughs) fast.”
Climate	**[ID #09]**“During the week, in this time of, of winter… just getting home already the, the night time, then no, uh, I, I don’t let them, them go outside.”
	**[ID #17]**“Then they go outside a little while to, to distract themselves outside. Later when it’s alright, the weather’s alright, yeah, or like we go out to ~ outside, just for a while, and then later everyone inside, when it’s alright already, ~ is over there, and then we go inside already. I mean, I say, “It’s just ~ if the weather is bad,” I say, well, I do say that when he says, “Let’s go play outside now!” I say, “No, mijo, if you go outside,” I tell him, “you’re going to get sick,” I say. “It’s raining, you’re going to get wet, and you’re going to get sick,” I tell him, “and you’re not going to want to be, to be sick.” He says, “No, well, no dad.” I say, “Good. We’ll go to ~ outside when ~ it’s a little while,” I tell him, and that’s it. “Let’s see, a ~ to watch movies or play something else here inside and, and there you go.” “Ah, alright, alright daddy.” And then, I mean, that is the common thing for me, that, like I tell him, I say, “If the weather is bad, well no, if the weather is good, yes.””
	**[ID #18]**“But it’s that ~ playing, so since it’s, it’s chilly, more than anything, right now, because it’s, well, he can’t be outside. And cold times or all that, well, no, it, they, like there’s a few weeks ago when illness came along with fever, bones, a virus. And because I didn’t want him to see ~ it because of the chilly outside, getting wet ~ and I put him inside to play there. That, it’s what we did. Or watching movies. Watch a movie, it would be him and my wife, eating there, watching movies at home. That is what I did.”
Safety	**[ID #10]**“Uh, when they see that I’m already outside, well, they want to go outside. I don’t like to leave them outside because, since it’s not fenced in or anything, it’s all open, so that it doesn’t happen that he, you just had a distracted [moment] and the kid is going to the road and well, there they do drive fast. And, uhm, that’s why just while I’m outside, then when I go inside, I bring them in with me. But I don’t like them, them to be there alone there.”
	**[ID #19]**“…there in the, in the, in the colonia where we live it’s very, uh, it’s very ugly, let’s say, I don’t know how to explain it to you, but it’s, it’s not, it’s very, it’s famous but not for, for good things but rather it’s famous for, for a lot, a lot of crime and things like that.”
	**[ID #06]**“The neighbors go outside too, the girls, and, and they’re there, there talking. And that the bikes, dang, they take off, back and forth, they’re there. Those have to be aware because they take off to the street. We already blocked the door, and, “Now go ahead, run there” “all you want, yeah.” Because they ran over one of my daughters a few years ago. And nothing happened to her, but yeah, she still did get hit. It threw her and since then I never let her go outside to, to the street, on the bike. Hey, people that don’t respect pass by. Hey, at forty miles [per hour] on these streets, how are you gonna go? ~ the kids, well, it was…my father in law, her grandpa, he’s taking care of them there, but she went outside, ran, went outside, to go around the, the street, well, didn’t he come and he hit her. And, and from that time on not anymore, and I tell my, my son, “Don’t let the kids ride their bikes on the street.” And then this girl too, that she wanted to take off, because on the street she says, “I can get flying.” “No,” I said. (All Laugh) “They’ll send you flying.” And I, and I tell her. And she says, “No, but I’m going to pay attention that nobody comes.” “Ask her,” I say, “ask my daughter,” I said. She’s twenty-eight already. And I say, “Ask her, when she was little, what happened.” And she says, “No, no, no, don’t even go close to the street,” she says,” “because some crazy [person] comes and he’ll run you over.” “Just like me.””
Relative deprivation	**[ID #16]**“It’s almost always like to the park or walking around there by the ducks that you said in the Edinburg [park], that’s where I go. I go there with them. You already see that you really distract yourself. I’ve been on the playsets sometimes with the child, the youngest little kid and the other one goes around fishing with me and there we go. And that is the, that is what I do, almost always. If for me, if I had I go fishing. Even if you catch one or not, sometimes you do catch some and you go, you still have fun, you spend time, you made time with everyone there, talking and, and it’s better, for them to be locked up, I’d better take them over there.”
	**[ID #22]**“And that is one of the problems that, that I find with what we have in, if you’re in the city, uh, even if it’s in the city there aren’t that many parks. There are very few, very few places where you can take the kids so that vas-, they really get distracted. And I have seen it in different parts of, of, of states. Different places, different cities and there are places that do put enough places so that the kids do activity. That’s something that I don’t see in this, in this area.”
Sedentary opportunities	**[ID #09]**“The only thing I say, “Look, you know what? Take everything away. Take it away.” I, that’s how I, I told her, “I did it like that with {child’s name} and he’s even cleaning his room.”… He did use to go out to play, but I did that so that he would clean his room, “But hey so that they go out, they’re, they’re very, they’re too overweight.” The doctor already told her too and I tell her, “Take away the TV, the games, everything, everything.” “At least two or three days.” “And then you put it back.” If they don’t do it, well I’ll take it away until they say that, that it comes from them, I mean no, because this one almost, he does turn on the TV but then he later turns it off and comes outside. “Daddy, I want to go outside again.” “Oh well, let’s go outside.””
	**[ID #03]**“On Sunday, like right now during the season, right now on Sunday it’s just waiting for the game and watching other players and finishing the chores in the house because once the game starts, that’s it. We sit down and that’s it. “
	**[ID #08]**“Giselle likes soccer, but, well 1090 sometimes I don’t have time to take her and I make her older brother take her. They go to the park, 1091 park that’s here, they play there. She likes soccer and, and l-, the {hobby}, like they say, of hers, 1092 is drawing. It’s what gives her ~ She draws, I don’t know how she does it to draw [like that], but 1093 she does draw well. (1 Laughs) But I don’t know she does it, but she taught herself and it’s what 1094 she does, draw and draw all day. Her free time is drawing.”
Access to transportation	**[ID #10]**“There are times that, the kids tell me ~ they, “Let’s go to the park.” “That’s fine.” And, well, uh, to not move the car, well, we go walking a little, and I get tired really fast from having to walk. My kids tell me, they go, “Dad…” they go, “you can’t stand anything.” I tell them, “No, well, it’s because I don’t really walk.” And then being in the park, and me without wanting they take me there to play there, there on the court, there for basketball. Well I get to the house all sore in the feet and all. I don’t want to get up the next day.”
**Ecological Model for Active Living–** ***Interpersonal***	
Community	**[ID #05]**“Since there are a lot of girls outside, she’s over there running. She’s, runni-, playing, running, and everything. And she sweats a lot. But she, she does a lot with, she’s with them every day. She’s playing, on the bike, running. She does do a lot, a lot of exercise. And that’s the activity that she does with them, playing and jumping.”
	**[ID #10]**“Well, the neighbors take care of us and, well, we… Because, well, they also have kids and sometimes they’re playing there in the street. And when we are outside we try to…or when mine sometimes go out before they’re told, ~ “Neighbor, your kid is already here.”
	**[ID #23]**“Uh, well, because, what happens is, since she’s a girl, and, and the, and the nextdoor neighbors are also girls, I do let her play because they’re girls. And, well, girls with girls. It’s logic there. And so the boys, well, with the boys, but when the neighbors’ girls are not there, well they play with my kids there all together. But no, I don’t have rules because I know they’re good, uh, they’re good girls. That we’ve known them for a long time there like neighbors. {So} we take care of each other all the time, we… We have a, good relationship with the neighbors there. We have been with them in the good times and the bad times, like we said, always. We’ve taken care of each other”
Family	**[ID #03]**“And, {so} on Sundays, uh, we go and buy like a pan dulce or barbacoa or something and we go to the grandma’s house and we spend time there because I don’t have much, much family here. I have some, but not as much as my wife, my wife has a lot of family. And almost all of them live there, in Mercedes, or they are close by, and they all go to the grandma’s house and they, they spend time there, they eat, or make food or… {So}, on Sunday, if, if we go (Mumbles) there, or if my mom, uh, picks up the kids, takes them to the flea market and only my wife and I stay, and the youngest one. And we go wander around or to walk around in a store or…we can see what, to see what we can do until the kids come, the other kids come back. And then, uhm, we get ready for the coming week because it goes by, the weekend goes by fast.”
	**[ID #10]**“Well for me Sunday because it is, uh, where we all get up together. We get ready and go to church and then from there, coming back to the house, well, then we spend the entire afternoon watching movies. Uh, making some sandwiches, there to watch the {movies} or some popcorn, right? But that is the, the day…for me that day is special because you are with family.”
Culture/norms	**[ID #21]***“*My wife, I say, “Let’s go do, do exercise” and she says, “No, well yes, let’s go out.” I mean, she is, she is backing me up, she’s helping me, she’s supporting me. And that also motivates you to, for you to, to also make a little more effort and it’s a little hard to make an effort. And, and, as I said, that almost makes the, the, the kids [do it] because I’ve seen it. If I’m outside and I’m doing it, all the kids come outside, but if I’m inside, uh, then they are inside. They don’t go outside. I mean, to motivate, uh, then you can tell that the dad is the motivation in the house because if, if I go outside to get in shape, man, all the herd comes with me, right, and if I go inside, they go inside. I mean, what am I trying to get across? That I’m like the teacher of, of my kids at home.”
	**[ID #03]***“*My son climbs and, and, and he exercises and he even, I have weights and sometimes, uh, he wants to pick up the weights, the smallest ones but he wants to and my son is strong. He does, like he’ll hang and he, and he, I don’t know what it’s called when you lift yourself like this. He does a lot of those and, and he does pushups and runs and I know that he is stronger and I think that maybe she, because she sees him do so much, she thinks that maybe like, she gets discouraged. “I can’t, I can’t do as much as he does, or I can’t run as fast.” But little by little, it’s just, I think that she sees her mom who, who walks more and exercises more, she sees that we, because I bought a table, I mean not a table, a, no, like to do, you lay down in the, uh, to do weights. {So} she sees that we use that, right? And, and that we do exercise. {So} she feels, she feels better, yes, she is doing more exercise. {So} just, I think it’s just that I have to continue to encourage her and showing her that everyone is different but that everyone can be healthy and be okay physically.”
	**[ID #05]**“But when I am with what I do, and there are times when we are, there outside of the house, like the ~ gentleman says, that we pick up everything that’s on the floor, the trash, everything, we clean the yard, clean the house. So we are, ~ all of us cleaning there. And, uh, they helped inside. I have three, three girls. ~ two older ones and a little one. The oldest help mom inside and the boy here outside with me, the other one. He’s thirteen already, {so} he’s picking up [stuff], he mows the lawn, cleaning. And he helps when I do jobs there at home, mechanic [work], he helps me.”
Parental & caregiving roles	**[ID #04]**“I’m not a father that you’d say is demanding. I like to give them advice because I imagine that with rules, they don’t listen to you and with advice they do. They trust you, they tell you what they do, they tell you everything. I try to, instead of being a, a father, I try to be a friend first. I want him to tell me his problems and I tell him mine. To tell him, “Don’t do this, don’t do that. Come on, I don’t want you to, in, where I can’t see you, I don’t want you at the neighbor’s house. Don’t do something that you shouldn’t do, climb the trees, because you can fall.” Like to see, to see the risks that, that he can have in, in a way, right? I try to take care of him.”
	**[ID #06]**“I mean, they feel good when, when their dad’s with them, “Let’s go play ball, let’s go play here, let’s go play this. Any game that they, be with them when, when, when, when they’re playing, right? So they feel, “Oh, yeah, look, my dad. He was playing with me.””
	**[ID #12]**“Well we’re the supporters (Chuckles) the supporters of ~ which is the best, right? Being healthy, right, for them. Right, because, you can imagine, if you leave, where are they going to stay? Right? It’s what I think about being healthy, right, to, to keep moving forward, right, with the, with the, your expenses, right, of the family and everything, right? Because, well you’re the one that, at least in my case and his, we’re the supporters.”
Occupational roles	**[ID #16]**“During the week I well I go to work, I go to work, right, I get home in the afternoon at five, six I get home and well sometimes I go to play soccer with the kids in the afternoon. Here at the park in the colonia. Or if now well I start watching movies with them or I play. I start playing the Nintendo with them there. And well you go like that, from going out like that it’s l-, really what I always do because I get home really late from work and now it’s already getting dark really early. And well I leave at five, six in the morning to work and I get home at six and the day that I get out early, well we go over there. Like I say, fishing or making time to go out and about. But almost always, always well really just getting home and playing with them there because there’s no time.”
	**[ID #23]**“For me, uh, a normal day is, I wake up at five in the morning. Uhm, I get ready for work and I work eight hours like a driver here in a do-, an adult center. {Day care, a-, a-, adult day care.}And, uhm, and I like to help older people. It’s my passion because I had grandparents and I always helped them, I used to help them to live, and, they are some of the reasons why I like this job a lot. And then I get out at three thirty in the afternoon and I get home and I like to do an activity, whether it’s something at home or, or to go don-, a sport with my son or to go to the store with my wife and, it’s part of a day, like, a complete day. And when it’s not a complete day, uhm, well, I am, uhm, either outside cleaning the yard.”
Household roles	**[ID #04]**“Anthony loves it, he loves being helpful. Uh, I, when I, when I bring groceries, he is the one that helps me to, to take all the groceries inside. He is the youngest and he is the most helpful out of, of all my, all my kids, he is the one that… I going around doing something, right? At home, like mechanical work or construction and he is the one that is right there helping me. So, he is the one that, yes. Yes, he is the helpful one of the, of the family.”
	**[ID #06]**“In, in the, in mine, uh, well at first, no, her mom wouldn’t make her do this, do that, I mean, it’s [about] teaching them, until they, uh, with me I tell her, “Look, that girl, you need to start teaching her.” “Look, uh, make her grab a broom and that she starts cleaning.” “So that she goes… Now,” I say, “you also have to teach her there in the kitchen how to wash the dishes there.” No, now she already, already, you tell her, “Come on, start sweeping.” She sweeps, cleans, everything, picks [stuff] up. And then, she tells her, “There are, uh, some dishes there, wash them.” A big amount, no, but one, so that she starts doing it. No, she does go and washes them and everything, yes, very well. Uhm, she picks up what, she puts her clothes away. I tell her, “You have to teach her, because,” “it’s something that she has to learn.”
Social support	**[ID #09]**“I bought all of them bikes….And now he does more, now they do want to ride (~ Laughs) And I also bought one for myself… now that they see now that I also ride my bike, well they, the younger kids too, “No, daddy I want [to]. Now the, the third, now he doesn’t, at first he didn’t know, only training wheels. Now he does now he’s caught on, now he just has one…. He thinks that I do hold him up but I let go and I tell him, “Should I take it off for you?” “No daddy, because I’ll fall.” “Man, well, it’s already free, look, it doesn’t, it doesn’t hold [you up] anymore,” but well, he thinks that it does.”
	**[ID #08]**“Yeah, every day ~ and she’s one of those [people] that isn’t still. She, “Hey let’s go, you told me that we were going to go.” Because you can’t promise her anything because she, she doesn’t get off you. With telling her, “I’m going to take you to {Chuck E. Cheese}.” You have to take her and he is the one that takes her, and her mom and her, her aunt, my son’s wife and… But not, just don’t tell her anything, don’t promise her, that’s why I tell me wife, “That’s why I don’t promise her anything, because if I promise her, I can’t finish it.” And I tell her, “Come,” just I, what I do, I just give her the money or if not my son pays for it. But I tell her, “I don’t promise her anything.” And this girl, because this girl doesn’t, she doesn’t forget anything. Yeah, yeah. But what she really likes is going around on, soccer and on her bicycle and her, drawing. It’s what she does in her free time.
	**[ID #10]**“Well, on one hand, like, well, there where I live I have neighbors, but, well, they don’t let them get together, I mean, they don’t let them go outside much. But, well, I do let mine outside there to play, if a boy comes over, whoever, on the contrary, I g-, go out, I, some juice so the kids can feel, feel comfortable, and then the kids that want to go then, “Where do they live?” “No, well, th-, there on the next street.” “Come here, I’ll take you because I don’t want anything to happen to you on the way.” {So}, then I take them and everything, but, (Pause) I don’t know.”
**Ecological Model for Active Living–** ***Intrapersonal (in Nvivo as “Participant”)***	
Recall	**[ID #09]**“I know that exercise, playing and all that is, is basic, but I also remember when I used to work in a big company, they made us exercise in the morning and some exercises. I mean, something that told us, “Look, do this. This helps so that for the body and digestion. I mean, so that you don’t d- [do], they did that so that you wouldn’t get a {cramp} at work. ~ no, no, look, we were really comfortable out working and no… well rested, on the contrary, for those that didn’t want to work, those ones were off hiding.”
	**[ID #14]**“Well I, I, I just [did] school too and then, a, first and second [grade], right, just second [grade]. Because I broke my foot. And I didn’t go to school anymore, I stayed at home there because I couldn’t walk, and it made me… lazy ~ uh, because I couldn’t walk well for a while, ~ I broke this foot playing there, I don’t know ~ playing at school. So then I broke my foot and didn’t, didn’t, didn’t go anymore, didn’t go any more to, to ~ because there they don’t have, like those, (1-‘Crutches.’)
Obligation	**[ID #17]**“I tell [my wife], like I, “You see it in my face when I get home from work to, I mean, irritated, tired, and at the same time I still have to take my responsibility as a father,” I tell her. “I still play and everything with, my children and everything. Why? Because it’s my responsibility as a father,” I tell her, “further along, they remember a father that was affectionate with them, that he was, he had time for them,”… I say, “It’s that, I get home tired from work, and all irritated,” “now, there are some plain days where yeah,” I say, “You know mijo, today I’m getting home, I can’t take it and I ~ just,” I say. I tell him, “forgive me,” I tell him, “I’m sorry, but, I just want to take a shower, have dinner,” I say, “and lie down,” I say, “I’m really tired,”…“But there are days when I do make an effort to, to focus, I mean, focus on them, after getting out, even though I’m tired and everything when I get home, I play with them for a while anyway and then that’s it.” But yeah, I mean, it’s your responsibility as a father.”
	**[ID #05]**“But yes, she tells me that if I feel, like, very, I’m putting pressure on myself, to work. But like you that, you start saying that you want, well, get ahead, right, well have more. But there are times that yeah, there are times when I can’t, because there’s a lot of work. I fall asleep, the exhaustion. Because what I do here is actual work, and then I get out and I do jobs here, and sometimes I do get tired. And yes, I do know that if I wanted to take, to be at home every, after five and to be with the family, but right now, if he is, but yes, I do tell you, like, like right know they went to look for me, but I said no. In reality Sundays not [so much] anymore. Yes, I am really fed up, I’m doing bad and…but yes, no, I do feel, like, a little, uhm, pressured, all of that.”
	**[Interviewer]**“*What is*,* what*,* uhm*,* would be the most amount [of time] that you spend playing with them in hours*,* or*,* or t-*,* or minutes?“* **[ID #14]**“Well, [for] me like an hour, I have seen ~ play an hour ~ Playing an hour like that with them. From then on then I get annoyed. “I don’t want to play anymore. Y’all play, I’m going to go inside now.” To watch TV again, or…”
Physical health	**[ID #21]**“What I’m most worried about is for the, for the boy’s weight, that’s motivating me to, to get in a better shape for his health. And for my health too because I already, sometimes I run and it’s like I get worked up, it already, like, I get out of breath… I am a little, my weight is a little, high, I think, maybe… I feel like worn out, I run a little and I feel like I’m out of breath and I can’t breathe. So that, it was helping me right now to start doing that activity because I’m getting worried about my older son’s health that he’s a little, he’s chubby, right? I want him to do, that he’s in better shape, that he loosens up, that he doesn’t struggle to walk because working out makes you more [physically] able.”
	**[ID #06]**“Yes, us too, we go out there, not every day, uh, twice per week, when it’s hot that it’s good to be there. Because also, uh, the girl, uhm, she suffers from bronchus, and in, uh, when it’s very fre-, the air is fresh or the weather, fresh like that. No, but when it’s hot, that the weather is fine, we go out to wander around. The bike, and walking. My wife takes a lap, two, three laps there. Two hours, we spend there and then we come back.”
	**[ID #10]**“There are times that, the kids tell me ~ they, “Let’s go to the park.” “That’s fine.” And, well, uh, to not move the car, well, we go walking a little, and I get tired really fast from having to walk. My kids tell me, they go, “Dad…” they go, “you can’t stand anything.” I tell them, “No, well, it’s because I don’t really walk.” And then being in the park, and me without wanting they take me there to play there, there on the court, there for basketball. Well I get to the house all sore in the feet and all. I don’t want to get up the next day.”
Mental health	**[ID #05]**“Well I say that because, I think that she’s well because she, it, she goes, since she has, she goes around really active, t-, she’s going around playing, she goes around, jumping and jumping, laughing and laughing, she runs around back and forth. No, it doesn’t seem to me that ~ like she looks like she’s bummed out, no. She’s always going around happy, laughing and laughing, watching TV, singing ~ like she’s singing”
	**[ID #06]**“It’s, the same thing, I mean, well, she… When children are healthy, they’re really active, really happy, going around. When they’re sick, then, they look ~ there, and she runs over here, and like she sings here, she sings there, and she goes around. She’s silent, sitting down there. You see right away when the person [is sick.] If even you, when you, no. Yeah.”
	**[ID #18]**“And the same thing, I, the same for me, I also, I mean, I take the boy and play soccer and, uh, there are times he goes around running or he’s a little kid and that, there I go, playing around there with him, wrestling there, he gets on top of me, everything. I mean, he says ~ he wanted to when I, him laughing, man, the rest… Imagine that for me s-, it’s something that I enjoy it, me, me, it makes me happy too to see him smile, I, seeing them smile and not see him sad. I mean, I want to see him al-, always smiling. Always smiling, all that like not being, uh, with his head down.“
Beliefs	**[ID #03]**“With my daughter… I think that, uh, she got her body from, from her mom, from her mom’s side of the family. But us too because when I was born, I was a big baby and she was really, really big. When I was born I weighed almost, I weighed twelve pounds. {So}, and she, she almost was, she almost hit ten pounds. {So} I know that she has always been a little fuller, a little bigger, a little more, uh, right…. She doesn’t look very, no, no, she’s not fat, but she looks a little fuller and she’s tall. And all the other girls at school, well, aside from the fact that they are almost like a year apart, yes, they are shorter. And, and I think that she kind of feels like she isn’t, like she isn’t, like exercise is not for her.”
	**[ID #03]**“You can tell because the kids, the, my daughter the one that is, in the middle, the oldest, the, the oldest of the girls, she is getting a little, she is getting a little chubbier, I think she has a body more like of, of the family of, of my wife. And, I tell her, we have to focus right now, uhm, on teaching her that it doesn’t matter how we, uh, we look, right? We always have to have lots of, uh, faith in our, in ourselves to, to know that we can change, that not because we are a little chubby, or overweight, it means that that’s how we are going to stay. And not just because she is, like my son is a little skinnier, it doesn’t, it doesn’t mean that you are going to be skinny your whole life. Later you will, you will, they will grow up and they see that we, uh, we exercise, that we go walking, we do things outside, they give, they like to go outside, and, and my daughter, the girl, we got a, a puppy and the puppy is growing up, so she goes outside and they feed her, and they give her water, and they play with her.”
**Additional themes**	
**Resilience**	**[ID #02]**“I do have time for them, in the evenings… I bought them their lights, because it’s always and night when they’re out in the street. Their little lights on their bicycles and I go with them and I go around [with] them. But, I do have time, but not enough.”
	**[ID #26]**“I get home tired, right? But you rest for a little bit and in the nights, right? I made them a soccer goal, right? But one of those made of pipes and everything. I started to play with my children, soccer, there at home and I prepared everything for them. And it makes you happy because in, it wasn’t my kids anymore, but the colonia started bring the children and the children arrived. And my neighbors got excited and, and even the men would come to play with us, and with their kids.”
	**[ID #09]**“On a normal day, that they, uh, I’m coming home like at, like at five, five thirty, uh. [When] I come home, they go out and, and do, like right now at this time, well, it’s, it’s early because it gets dark earlier. Already by six it’s already almost…and sometimes, uh, I let them until eight, but I put a lightbulb outside for them there because they don’t want to come in.”
	**[ID #20]**“Well, okay, you do feel, you do feel bad, right, or sad or I’m not sure what it’s called, right, because, well, as a matter of fact, uhm, uh, you should dedicate time to the kids, right, to the, to your wife and to the kids but, like I said, well, uhm, you really just live fo-, I mean, well, to cover the expenses, you need to work. And, well, at work, well, you’re not going to set your… Well, uh, in my case, right, that I’m an employee, well, I can’t set my own hours. So, I get out late, there isn’t any time to, to, to do anything.
**Meaning/** **Experience of Physical Activity**	**Outcome**
Intrapersonal/participant	**[ID #03]**“There are times that, like, I exercise with him I make him, him, I tell him, I make him write with both hands, to throw with both of them, to, how do you say it? To use one hand, I’ll close his eyes, I tie one of his hands. I make him use both, I do make him do more because he does, he does want to, like it calls him more, certain sports. All the three that I like, he likes and he wants, and he wants more and I tell him, “I can help you out with these three because I know these three, the, if you want {soccer} or if you want baseball, well, I can learn them. I don’t know anything about that one but if I learn and if you want to know, or if you want learn from that one, you want to play, that’s fine.” I don’t want him, I don’t want them to choose something just because I like it. I have a passion for these three, but I also like everything else. It’s just that I never, no, I mean, I realized that I have talent for these three and for the others not so much {so} I never pursued them, like they say, no, no. But if they like it, whatever it is, I put a-, aside what, what I want to learn to teach them something different.”
	**[ID #08]**“I’ve always been a professional soccer player, that’s why my daughter really likes it. Because I would always play soccer and I was good at playing.”
Interpersonal	**[ID #04]**“I mean, he goes out with his, with his friends to play, he loves to play with them, running and, and he does this and he does that, they bounce and jump. There with them playing, right? And he likes sports, he likes to hit the ball and he likes exercise. I mean, he started doing weights and this and that. I mean, he is a very active kid, he does like exercise a lot and like I said, the most, the exercise that he does the most, right? That doesn’t look like it but it is playing. Play with his friends, I mean, it’s the best exercise a kid can have. Because, well, he does it with joy and with energy and he is, he is exercising. Even if he doesn’t see it as exercise, it is exercise. And even for yourself, too, right?”
	**[ID #05]**“Well she is, since there are a lot of girls outside, she’s over there running. She’s, runni-, playing, running, and everything. And she sweats a lot. But she, she does a lot with, she’s with them every day. She’s playing, on the bike, running. She does do a lot, a lot of exercise. And that’s the, the activity that she does with them, playing and jumping, she jumps a lot too. She’s running, she’s riding her bike.”
	**[ID #15]**“Well, my son doesn’t like any sports. Nothing. He’s eight years old, I’ve said, at my house he doesn’t do anything physical. I mean, sometimes he’s, he’s on the swings, right, just on the swings. And like, for him to go play with his friends, nothing physical, I mean nothing like running, not even playing soccer. I’ve told him, “Do you want to play soccer?” and he tells me, “Yeah, I do want to play,” and I buy him his tennis shoes, his tennis shoes and his ball. And, “I’m going to teach you how to play to put you on a team.” Man, the next week he drops it all. “What do you want to do?” “I don’t know, well, uh…” he starts, “I like baseball.” He comes out with that, he comes out with that. “Well, I’m going to buy you a {bat} and to play baseball. A {bat}, a ball and glove.” And I’m there looking for a, and I start there too right there at home, in the backyard to, to play with him. And he drops it. Not that one either, nothing. He doesn’t want it, he doesn’t like anything. I tell him, “What sport to you like?” and then he gets out, he comes out with another one, with another one.”

Public infrastructure investments were also discussed as impacting PA, in both indoor and outdoor spaces (Table 2). There was a desire for additional PA resources for people of all ages in *colonias*:

*That*,* what’s more*,* even in Mexico that is a really very poor place*,* you still see {soccer} fields. Places where you can get distracted. You see a lot*,* a lot of activity from the kids… compared to what we see here*,* it’s nothing not even close.*

*They want to play a sport*,* they want to play {soccer}*,* they want to play {basketball}*,* that park is even more useful for them*,* but there aren’t enough for everyone.*.

Strategies to overcome poor investment in public infrastructure include reserving time to travel longer distances to parks or recreational facilities on the weekend and prioritizing PA in/around the home (Table 2). Fathers emphasized that increasing the number of neighborhood parks would not only make it easier to support their children’s physical activity but also enhance overall quality of life in colonias.

Law enforcement policies and funding were another barrier to PA mentioned by participants. This theme was relevant when fathers discussed their immigration status, given that immigration enforcement was a barrier to traveling to different PA opportunities out of fear of being stopped, questioned, incarcerated, and/or deported (see Table 2). Law enforcement also reportedly frequently surveilled participants’ neighborhoods:

*The police pass by alot*,* and one time they passed and saw a ton of kids… Where I used to live and they even came down on me*,* it all came down on me. They even wanted to take my children away because they were out playing on the side like that.*

*I worked it out, we all went to Juarez, no ~ a year ago. That was a year ago, I sorted out their papers.… I couldn’t take my nephew with me of, he doesn’t have papers. He’s here right now, well he’s just in school. But my wife wants to get him a permiso but there wasn’t, wasn’t time and I just went like that. But I did take him, {I mean}, we do, do go out and around, and when there’s a chance we go anywhere [around] here.*

Strategies to overcome these barriers to PA included refraining from longer-distance travel, pursuing citizenship for family members, and increasing parental supervision for outdoor PA (Table 2).

Low access to jobs was a major barrier to PA. Many participants mentioned working low-paying jobs with long work hours, manual labor, or seasonal travel commitments:

I worked years ago, I worked when I was in the {Border Patrol}. The ~ was 24 h that I had to work and sometimes I didn’t get [home]. And sometimes I got used to not even see my wife, or the kids because, well, there wasn’t time, they went to school. But at the same time you lose that affection with the kids, you lose that opportunity to get them, to take them to play or to eat together and all that.

Given that fathers felt a responsibility to provide for their families, strategies to overcome long work hours to engage in PA included installing lights for outdoor PA or engaging in indoor play, or resorting to sedentary activities with their children (Table 2).

Fathers mentioned school policies that made it difficult for children to miss school or caused them to miss PA opportunities after school (Table 2). Participants also discussed the importance of schools for providing PA opportunities:

I remember that when they had the {open gym}, they would open the school gym as well and, I didn’t, in reality I don’t know why they don’t do it anymore, because it’s a public school. They should do it by law, but they don’t, they don’t do it.

Fathers did not mention many strategies to overcome school policies that inhibited PA for their children (Table 2).

### Environmental influences on PA

Access to environmental PA resources, and particularly outdoor PA opportunities, was mentioned as important for promoting or inhibiting PA.

To start, neighborhood resources and PA opportunities were mentioned often and mostly included streets, sidewalks, neighbors houses, local parks, and nearby sports facilities. Parks were identified as places that support culturally relevant activities, such as football, soccer, and fishing. Fathers viewed taking their children to parks or other public spaces as important opportunities for both bonding and PA. Neighborhood spaces facilitated social interaction between children, and were places where parents shared the responsibilities of supervising play:

When we used to live in the apartments, there were a lot of, like you say, there were a lot of kids that went and played, and many of those kids were the kind that the moms or dads didn’t take care of. So since it is an apartment and it’s enclosed, well, the whole afternoon since they get out of school, they are up and down, and it gets dark, and those kids are still outside.

Strategies to facilitate PA in neighborhood spaces included coordinating with neighbors to provide regular parental supervision, providing opportunities for children to play with one another, and providing transportation to local parks and recreational facilities (Table 2). Environmental resources/opportunities around the home or yard were also seen as.

crucial for PA. Fathers mentioned a high need for parental social support and supervision when children were playing outside the home. Fathers also participated in housework with their children in the yard (Table 2). It was mentioned that sons were more frequently asked to do outdoor, manual labor or housework (e.g., mowing the lawn) compared to daughters who assisted more with indoor chores:

Yes, we get [home] later at night, because, the night falls… We pick up everything that’s on the floor, the trash, everything, we clean the yard, clean the house. So we are, ~ all of us cleaning there. And they helped inside. I have three girls. ~ two older ones and a little one. The oldest help mom inside and the boy here outside with me, the other one. He’s thirteen already, {so} he’s picking up [stuff], he mows the lawn, cleaning. And he helps when I do jobs there at home, mechanic [work].

Strategies to facilitate PA in outdoor environments included buying or building/creating PA equipment (e.g., soccer nets), installing lighting, providing supervision and social support for outdoor PA, and creating rules meant to ensure safety (e.g., stay away from traffic, avoid strangers) (Table 2).

Environmental resources/opportunities inside the home were mentioned as important for facilitating PA especially when it is dark outside, children lacked supervision, or outdoor environments were unsafe (Table 2):

Because when my wife is there alone, well she says, “Man, to have them there outside.” “Some take off that way to the street and others the other way and I can’t watch them anymore.” And that’s why no, she doesn’t take them out, but there inside the house keep in mind that they have a track. From some rooms to another and then back.

Strategies to facilitate indoor PA included buying PA equipment (e.g., active video games, dance videos), assigning active housework/chores, allowing children to invite friends over, or creating physical space for PA (e.g., indoor track, playroom) (Table 2).

Sedentary opportunities were commonly discussed. Participants discussed some sedentary family bonding activities, including going to the movies or watching television (Table 2). Some fathers also discussed how their children preferred sedentary activities over PA:

She likes soccer and, and l-, the {hobby}, like they say, of hers, is drawing… But I don’t know she does it, but she taught herself and it’s what she does, draw and draw all day. Her free time is drawing.

Strategies to avoid excess sedentary behavior and promote PA included restricting screen time for children and encouraging children to spend time outdoors (Table 2).

### Interpersonal influences on PA

Family was an important factor influencing father and child PA, and included factors like family structure and size, extended family dynamics, routines, and the importance of family. Strategies to promote PA amid family dynamics included incorporating PA into family routines or providing PA opportunities that appealed to the interests of all family members (Table 2).

Parental and caregiving roles included participant perceptions of their roles as parents (e.g., modeling, quality time, teaching, discipline) and caregivers (e.g., caring for grandparents, nephews/nieces). Fathers mentioned that it was their responsibility to provide PA opportunities, which seemed to be a prominent motivator for maintaining regular PA (Table 2):

I mean, they feel good when, when their dad’s with them, “Let’s go play ball, let’s go play here, let’s go play this. Any game that they, be with them when they’re playing, right? So they feel, “Oh, yeah, look, my dad. He was playing with me.

Strategies to promote PA as part of parental roles included using PA to bond with children, assisting their children to explore PA that appeals to them, and providing structure and rules for their children as they engaged in PA (Table 2).

Occupation roles were mentioned as a major barrier to PA with children. Fathers reported long work hours and inconsistent work schedules, which often caused them to get home after dark (Table 2). They also noted that jobs requiring physical labor caused them to be tired and have less capacity to engage in PA with their children upon returning home:

I get home really late from work and now it’s already getting dark really early. And well I leave at five, six in the morning to work and I get home at six and the day that I get out early, well we go over there. Like I say, fishing or making time to go out and about. But almost always, always well really just getting home and playing with them there because there’s no time.

Strategies discussed by fathers to overcome occupational barriers to engaging in PA with their children included making sure to make time for PA after work (even in the dark) and spending days off at parks or other PA destinations (Table 2).

Household roles were mentioned by fathers as often entailing PA, taking place outdoors, and involving their children. Fathers expressed certain gender norms around the types of household tasks they were responsible for and made it a priority to teach their children these tasks (Table 2):

He is the youngest and he is the most helpful out of, all my kids, he is the one that… I going around doing something, right? At home, like mechanical work or construction and he is the one that is right there helping me.

Strategies to promote PA while accomplishing household roles included involving children in the tasks and using active household tasks as a chance to bond with their children (Table 2).

Culture and norms were important for PA according to fathers in this study. This included culturally relevant PA (e.g., football/soccer, fishing), gender norms for PA and parenting, cultural norms around family gatherings, and community social cohesion (Table 2).

You can tell that the dad is the motivation in the house because if I go outside to get in shape, man, all the herd comes with me, right, and if I go inside, they go inside. I mean, what am I trying to get across? That I’m like the teacher of, of my kids at home.

Fathers identified strategies to promote PA that aligned with cultural norms including making time to teach their children soccer or other culturally relevant pastimes, taking on PA promotion as part of their role as fathers, and spending time with extended family (Table 2).

Community was an important facilitator for PA among children and provided important support for fathers as they promoted PA in their families. This was especially important when fathers did not have the social support within their family unit to accomplish certain tasks or facilitate PA among their children. PA often involved supervision, indicating the importance of adult community members to provide this supervision when fathers could not do so (Table 2). For this reason, social support codes often overlapped with community and parental roles. Social support was also necessary for engaging in PA, traveling to PA destinations (e.g., parks, soccer fields), providing PA resources, and promoting PA among children (Table 2).

### Intrapersonal influences on PA

Obligation was coded when fathers expressed feelings or emotions about expectations or roles that forced them to put aside personal needs and interests. This often meant losing opportunities for rest and personal PA opportunities, as well as missing job opportunities (Table 2). Sense of obligation also intersected with gender norms, as participants felt that self-sacrifice was part of their responsibility as fathers:

I tell [my wife], like I, “You see it in my face when I get home from work to, I mean, irritated, tired, and at the same time I still have to take my responsibility as a father,” I tell her. “I still play and everything with, my children and everything. Why? Because it’s my responsibility as a father.

Strategies to overcome this sense of obligation to promote PA included reconceptualizing their obligation to spend time with their children to lighten their mood or make time for themselves (Table 2).

Fathers discussed their beliefs about PA, including its importance and health benefits. These beliefs were often shaped by their own experiences and health status, and included ideas on the relationship between PA, healthy eating, and weight status or health. Many fathers expressed beliefs that their children inherited their own proclivity for PA, sport, or their body shape, or were influenced by their physical inactivity.

Physical and mental health were often mentioned in relation to PA among fathers and children, and most participants believed PA was a key determinant of physical and mental well-being. Physical and mental health symptoms were also mentioned as barriers to maintaining PA or being active with their children (Table 2). Fathers promoted PA among their children even if they couldn’t participate in or use PA to improve their own health. When fathers mentioned their children having physical health constraints related to PA, they discussed helping their children overcome them through motivation or adaptations (Table 2).

Recall included past experiences that shaped the way fathers thought about PA and promoted PA among their children. Fathers mostly focused on policies or environmental features that they thought positively influenced PA or discussed negative health behaviors they needed to overcome in their own lives to become good role models to their children.

They used to have a program at school before, in {high school} where they would open the {gym} and they could go play basketball and on one hand it was good because when it rained, well, I remember that in, with, at school we wanted to go to the well, in fact, we always had a park,but when they opened the {gym},well we went… in {high school} I played basketball,I played soccer,(Laughs) everything. I started,I started in {weights} too but,man! I was really skinny.

Recalling past experiences helped fathers both develop strategies to promote PA and remember ways they previously overcame barriers to positive health behaviors (Table 2).

### Resilience

Resilience describes strategies for overcoming barriers or taking advantage of PA opportunities. Fathers faced barriers to PA but developed creative solutions to facilitate PA within their families (Table 2):

I get home tired, right? But you rest for a little bit and in the nights, right? I made them a soccer goal, right? But one of those made of pipes and everything. I started to play with my children, soccer, there at home and I prepared everything for them. And it makes you happy because in, it wasn’t my kids anymore, but the colonia started bring the children and the children arrived. And my neighbors got excited and, and even the men would come to play with us, and with their kids.

Other examples of resiliency included:

- Installing lights on bikes or for the yard to facilitate PA after dark.
- Making time to go to parks or beaches further from home.
- Creating makeshift PA opportunities around the home.
- Facilitate indoor PA after dark, by creating indoor tracks or other PA opportunities.
- Building social connections in their community.
- Reserving weekend or morning time for engaging in PA with their children, when work hours are long.
- Develop strategies to help their children with special needs to participate in PA.

### Meaning and experiences of PA

Participants in this study drew meaning from their own and their children’s participation in PA, and the meaning of PA acted as a reinforcement. Fathers valued PA as a tool for personal development and improved physical and mental health (Table 2). Fathers also described engaging in PA to develop physically and improve their health and strength, which was a motivator for their children to engage in regular PA (Table 2). Fathers most often valued PA as an opportunity to spend time with friends and family. They believed promoting PA was an essential part of their role as fathers, and most of their quality time spent with their family was doing PA (Table 2).

*And he likes sports*,* he likes to hit the ball and he likes exercise. I mean*,* he started doing weights and this and that. I mean*,* he is a very active kid*,* he does like exercise a lot and like I said*,* the most*,* the exercise that he does the most*,* right? That doesn’t look like it but it is playing. Play with his friends*,* I mean*,* it’s the best exercise a kid can have. Because*,* well*,* he does it with joy and with energy and he is*,* he is exercising.*

## Discussion

This study explored multi-level influences on PA, among Mexican-heritage fathers and their children living in a functionally rural community along the Texas-Mexico border. Factors perceived as promoting or inhibiting PA aligned with the social-ecological framework on the policy, environmental, interpersonal, and intrapersonal levels. Importantly, fathers communicated the importance of cultural norms for promoting PA. There were clear gender expectations for PA and housework, close-knit community structures that provided social support for child PA, and paternal roles to participate in culturally significant PA (e.g., soccer, fishing). Results also revealed two additional themes: resilience and the meaning of PA. Fathers felt it was their role to overcome barriers to PA, like long work hours and unsafe neighborhood environments, to facilitate PA and bond with their children.

While existing research mostly focuses on individual-level factors related to PA outcomes among underserved community members, this study revealed that Mexican-heritage fathers in the study were aware of both proximal and distal factors that could be modified to improve family PA outcomes. Amid a persistent lack of investment in and disenfranchisement of border towns like *colonias* [31–33], fathers in this study were resilient and prioritized PA. Participants mentioned a myriad of strategies to overcome these place-based barriers, including introducing PA resources at home (e.g., makeshift backyard soccer nets), overcoming occupational barriers (e.g., installing lights on bikes or in the yard), or making time to travel to PA opportunities (e.g., weekend trips to play soccer or fish). This article should serve as a call to action for policymakers serving *colonias* and similar border towns to address low access to recreational opportunities, unsafe neighborhood conditions, and long work hours.

Our research supports and adds to past qualitative and quantitative research drawing on fathers’ perceptions of PA opportunities and barriers that influence child PA. Qualitative research on socioeconomically diverse fathers reported paternal beliefs that child PA could be increased by providing supportive PA environments and parental role modeling of PA behaviors [16]. Quantitative research supports these findings, showing that father-child time doing sports, neighborhood environment, and parental perceptions of neighborhood environments significantly impact child PA in a nationally representative sample [18]. This study similarly highlighted the importance of neighborhood and home environments for promoting PA among children in *colonias*. Additional research supports our findings highlighting the importance of cultural norms, specifically related to gendered PA and paternal roles [64]. Our findings highlighted the actual lived experiences of fathers and their children in dealing with neighborhood safety and resource access issues, confirming findings from past research in *colonias* that documented barriers to active transportation and poor pedestrian environments [36]. Our findings also align with research highlighting occupational barriers to promoting child PA among Mexican-heritage fathers in rural areas [19]. Qualitative research shows that, despite fathers believing that PA was necessary for their children, long and laborious work hours made it difficult to model PA or participate in PA with their children [19, 65]. In addition, research shows that Mexican-heritage fathers feel a responsibility to provide financial security to their family, and this can be a barrier to family PA [66].

### Implications

This study has implications for future research. First, research is needed to quantitatively assess the relationship between multi-level PA determinants identified in this qualitative study and PA behaviors among children and families in *colonias*. Similarly, research is needed to explore the importance of resilience and meaning-making in allowing families to overcome multi-level barriers to PA in under-resourced settings. Next, similar research should be replicated among fathers and families in more diverse locations along the US border with Mexico to understand the most consistent barriers and facilitators for PA in under-resourced and functionally rural border communities. Finally, this study draws attention to the issue of Mexican heritage families facing long work hours with low pay. This may be at least partly due to immigration policies that limit job opportunities and facilitate worker exploitation [67–69]. Future research should assess the relationship between occupational constraints and health outcomes (e.g., type 2 diabetes, pain).

Our research also has implications for public health practice. This study identified policy and environmental factors that promote or inhibit PA in colonias. Local *promotoras* and other health workers in colonias can use our findings to advocate for targeted policy and environmental changes that support community health. Policymakers should prioritize infrastructure improvements in unincorporated communities like San Carlos, Texas, including constructing continuous sidewalks, installing street lighting, and implementing traffic-calming measures (e.g., speed bumps, crosswalks, signage) to ensure pedestrian safety. Local governments can collaborate with county agencies and regional planning organizations to allocate transportation and infrastructure funds specifically for underserved *colonias.* Additionally, zoning and land-use policies can be revised to ensure access to green spaces and recreational facilities within walking distance of homes. Community-based initiatives should also include outreach strategies tailored to Mexican-heritage families—especially fathers—such as culturally relevant messaging and programs that accommodate work schedules and family responsibilities. These findings have already informed the design of the USDA-funded *Salud para Usted y Su Familia* (Health for You and Your Family) program [52], which incorporates family-centered approaches to PA promotion.

### Limitations and strengths

This study has limitations that should be considered. First, this study recruited participants in *colonias* and is not generalizable to all border towns or Mexican-heritage people living in the US. The dyadic interviews used for this study only engaged fathers, and results may not reflect the experiences of their entire family or mothers. Next, fathers interviewed for this study were individuals who were willing to participate in a PA and nutrition program so participants in this study may be more motivated toward healthy behaviors and may have more access to certain resources (e.g., transportation, internet) than community members who did not participate in the study. This may explain why transportation barriers were rarely mentioned by participants when they discussed visiting PA facilities in *colonias*. Next, *promotoras* led interviews with fathers which may have introduced social desirability bias. Nonetheless, *promotoras* are trusted health promotion specialists and were instrumental in leading recruitment. Finally, the interview guide was developed to inform the *¡Haz Espacio Para Papi!* pilot program [53, 56] and ask about barriers and facilitators to PA and healthy eating generally. Therefore, our results may underestimate the influence of more distal factors, like policy determinants for PA, given that participants were not explicitly asked about this. This was reflected in our results, given that interviews had less discussion of policy determinants of PA.

This study also has important strengths. This research used dyadic interviews to allow for rich dialogue between fathers in this study about PA determinants. The dyadic interview strategy was successful in allowing participants to build off of each other’s ideas, and this study demonstrated that dyadic interviews with father-father pairs should be used in the future to gain insight into certain health phenomena within family units [41]. Adding to this, the project team included *promotoras de salud* to facilitate recruitment and dyadic interviews. Next, the coding and analyses for this qualitative research were collaborative and iterative. Our team engaged in peer review for maximum quality, and experts in the field of PA, health promotion, and multi-level determinants of health behavior contributed to our analyses, results, and discussion. This qualitative research was also grounded in theory to ensure results reflected well-known multi-level determinants of PA that would have otherwise been overlooked in coding processes.

## Conclusion

Using the social-ecological model as a framework for this qualitative study, we identified understudied contextual factors that influence father and child PA. Findings from this study highlight a clear need for systems and policy-level strategies to address occupational and environmental barriers to father and child PA in *colonias*. Nonetheless, fathers in this study were resilient and employed many strategies to overcome their low access to PA resources. Especially in low-resource, often forgotten communities along the Texas-Mexico border, it is important to identify modifiable barriers to PA that can affect population health.

## Supplementary Information

Below is the link to the electronic supplementary material.

Supplementary Material 1

Supplementary Material 2

## Data Availability

The data that support the findings of this study are available on request from the corresponding author, MEW. The data are not publicly available due to their containing information that could compromise the privacy of research participants.
