# Supplementary material for: Multilevel determinants of paternal and child physical activity: qualitative research using dyadic interviews among Mexican heritage fathers living near the Texas-Mexico border
Source: BMC Public Health. 2025 Aug 16;25:2814. doi: 10.1186/s12889-025-23956-x (PMC12357402; doi:10.1186/s12889-025-23956-x)
Supplement: Supplementary file 1 — Supplementary Material 1 [file 12889_2025_23956_MOESM1_ESM.pdf]

Fathers Dyadic Interview

Discussion Guide
(Draft version 07.11.16)

M = Moderator **P1 = Participant 1** **P2 = Participant 2**

M: Just to start with, we ask you to introduce yourselves, your first names, what you do for a living, tell us about your children/family, something like that for the "get acquainted part" and that will also help us capture this on the recording so when we go to type it up, we'll know which of you is which, which should be obvious.

P1:

P2:

M1: Since you've become a father, what has changed in your life?

General probes:

- Who has an example or story?
- Think about any other changes that make a difference for you.
- Compare stories; expand on ideas.

M2: What is being a father like?

General probes:

- Anything that makes a difference for you.
- Different roles.
- I'd like to hear about some of your experiences; so use examples and stories.

M3: In what ways are your child/children's food choices and eating patterns determined by you and others in your family?

General probes:

- Types of foods or snacks you want in the home
- Eating or cooking activities that you are part of
- Time when you and your wife disagreed on what child/children eat for meals or snacks
- Influence of other children or adults in your home
- Compare and expand

M4: In what ways are your child/children's playing and physical activities determined by you and others in the family?

General probes:

- Where they play
- With whom they play
- When they play
- Physical activities that you do with your child/children
- Difficulty being physically active with children
- Compare and expand

M5: What do you think about your child/children's eating and snacking habits?

General probes:

- Let's hear more about your reactions and feelings
- Compare and expand

M6: What do you think about your child/children's physical activity?

General probes:

- Let's hear more about your reactions and feelings
- Compare and expand

M7: If we developed a family program to improve eating and increase physical activity among your children, would you want to be part of it?

General probes:

- What do you think should be included in a program?
- Let's hear about how the entire family could be involved.
- Help or prevent
  - What can we do to work around your schedule?

M8: If your child was completely healthy, what would that look like?

Follow-up:

- What would you be doing?
- What would your child be doing?
- What would other members of your family be doing?
- How would you know that your child(ren) are completely healthy?
