# Supplementary material for: Multilevel determinants of paternal and child physical activity: qualitative research using dyadic interviews among Mexican heritage fathers living near the Texas-Mexico border
Source: BMC Public Health. 2025 Aug 16;25:2814. doi: 10.1186/s12889-025-23956-x (PMC12357402; doi:10.1186/s12889-025-23956-x)
Supplement: Supplementary file 2 — Supplementary Material 2 [file 12889_2025_23956_MOESM2_ESM.pdf]

Entrevista Diádica de Padres

Guía de Discusión
(Versión preliminar 07.11.16)

M = Moderador **P1 = Participante 1** **P2 = Participante 2**

M: Solemente para empezar, les pedimos que se introduzcan, sus nombres, a qué se dedican, que nos digan de sus hijos/familia, algo por el estilo para la parte de “darnos a conocer” y eso también nos ayudará a capturar esto en la grabación para cuando lo tengamos que transcribir, sabremos quién es quién, que debería de ser obvio.

P1:

P2:

M1: Desde que se convirtió en padre, ¿qué ha cambiado en su vida?

Sondeos generales:
- ¿Quién tiene un ejemplo o una historia?
- Piense en cualquier otro cambio que marque la diferencia para usted.
- Comparar historias; ampliar ideas.

M2: ¿Cómo es ser padre?

Sondeos generales:
- Cualquier cosa que hace una diferencia para usted.
- Diferentes papeles.
- Me gustaría escuchar acerca de algunas de sus experiencias; así que usen ejemplos e historias.

M3: ¿De qué maneras las elecciones de comida y hábitos alimenticios de su/sus hijo/s son determinadas por usted y otros miembros de su familia?

Sondeos generales:
- Tipos de comidas y *snacks* que quiere en su casa.
- Actividades de comer o cocinar de las cuales usted es parte.
- Ocasiones en las que usted y su esposa estaban en desacuerdo sobre lo que el/la niño/a comen de comidas o *snacks.*
- Influencia de otros niños o adultos en su casa.
- Comparar y ampliar.

M4: ¿De qué maneras las actividades de juego y actividad física de su/sus hijo/s son determinadas por usted y otros miembros de la familia?

Sondeos generales:

- En dónde juegan
- Con quién juegan
- Cuándo juegan
- Actividades físicas que usted hace con su/sus hijo/s
- Dificultad siendo físicamente activo con sus hijos
- Comparar y ampliar

M5: ¿Qué piensa acerca de los hábitos alimenticios y *snacks de* su/s hijo/s?

Sondeos generales:

- Vamos a escuchar más acerca de sus reacciones y sentimientos
- Comparar y ampliar

M6: ¿Qué piensa acerca de la actividad física de su/s hijo/s?

Sondeos generales:

- Vamos a escuchar más acerca de sus reacciones y sentimientos
- Comparar y ampliar

M7: Si desarrollamos un programa familiar para mejorar la alimentación y aumentar la actividad física entre sus hijos, ¿a usted le gustaría ser parte de esto?

Sondeos generales:

- ¿Qué piensa que se debería de incluir en este programa?
- Vamos a escuchar de cómo toda la familia puede involucrarse.
- Ayuda o prevención
    - ¿Qué podemos hacer para acomodar su horario?

M8: Si su hijo/a estuviera completamente sano/a, ¿cómo se miraría eso?

Seguimiento:

- ¿Qué estaría haciendo usted?
- ¿Qué estaría haciendo su hijo/a?
- ¿Qué estarían haciendo los otros miembros de la familia?
- ¿Cómo sabría usted si su/sus hijo/s están completamente sano/s?
